# Multiple Sclerosis Treatments Affect Monocyte-Derived Microvesicle Production

**DOI:** 10.3389/fneur.2017.00422

**Published:** 2017-08-22

**Authors:** Maria Blonda, Antonella Amoruso, Roberta Grasso, Valeria Di Francescantonio, Carlo Avolio

**Affiliations:** ^1^Department of Medical and Surgical Sciences, University of Foggia, Foggia, Italy

**Keywords:** multiple sclerosis, microvesicles, monocytes, disease modifying drugs, Inferferon beta, Teriflunomide, Fingolimod

## Abstract

Microvesicles (MVs) are released by immune cells especially of the myeloid lineage upon stimulation with ATP on its cognate receptor P2X7, both in physiological and pathological conditions. In multiple sclerosis (MS) the role of MVs remains little investigated. We aimed to compare the release of MVs in peripheral blood monocytes from MS patients with healthy donors (HDs) and to see how current MS treatment may affect such a production. We also assessed the treatment effect on M1 and M2 monocyte polarization and on the inflammasome components. Spectrophotometric quantification was performed to compare monocyte-derived MVs from 20 untreated relapsing-remitting MS patients and 20 HDs and to evaluate the effect of different treatments. Subgroups of nine interferon-beta and of five teriflunomide-treated MS patients were evaluated at baseline and after 2, 6, and 12 months of treatment. Six MS patients taking Fingolimod, after switching from a first-line therapy, were included in the study and analyzed only at 12 months of treatment. MVs analysis revealed that monocytes from MS patients produced vesicles in higher amounts than controls. All treatments reduced vesicle production but only teriflunomide was associated with a downregulation of purinergic P2X7 receptor and inflammasome components expression. The therapies modulated mRNA expression of both M1 and M2 monocyte markers. Our results, suggesting new molecular targets for drugs currently used in MS, may potentially provide useful novel evidence to approach the disease.

## Introduction

Multiple sclerosis (MS) is an inflammatory disease that affects the central nervous system (CNS) mainly in young adults with a complex predisposing genetic trait and plausibly needs a triggering environmental insult (e.g., a viral infection) to promote the disease (1). Early diagnosis in MS is important in order to initiate the opportune treatment, thus limiting the irreversible neurological damage that leads to severe clinical disabilities. To this aim, a possible identification of biomarkers characterizing the different MS phenotypes or indicative of treatment response is crucial, therefore representing a relevant target for research efforts.

In recent years, increasing evidence has shown the role of extracellular vesicles (EVs) as mediators of several cellular functions, including cell–cell contact and transfer of secreted molecules (2). EVs are membranous vesicles released by most cells (3); although full agreement on EV identification and contents is still lacking, they can be broadly classified as follows: apoptotic bodies (800–5,000 nm in diameter); large microvesicles (MVs) or ectosomes (MVs, 50–1,000 nm in diameter), produced through direct budding of small cytoplasmatic protrusions; and exosomes (EXOs, 40–100 nm in diameter) that arise in the endocytic pathway (2). Several studies report the importance of EVs (mainly MVs and EXOs) in CNS activities, e.g., in neuronal signaling and immunological responses. Indeed, glia and neurons secrete EVs and the recent literature indicates that intercellular communication by EVs has versatile functional impact in CNS homeostasis, including myelin formation, metabolic support, and immune defense (4).

Besides physiological processes, EVs have been shown to be involved in several neurological diseases as cargo of specific potentially detrimental molecules (5). Several studies reported alterations in the number and function of circulating EVs in Alzheimer’s and Parkinson’s diseases, epilepsy, stroke, and traumatic brain injury (5). In the pathogenesis of MS, EVs show both protective (e.g., inducing the maturation and migration of oligodendrocyte precursor cells) and damaging functions. As a matter of fact, they seem to be involved during relapses by activating cells, by leading to migration through the blood–brain barrier and spreading phlogosis in the CNS (6). Indeed, it has recently been demonstrated that the amount of microglial EVs in human cerebrospinal fluid (CSF) is higher in patients presenting a clinical isolated syndrome (CIS) suggestive of MS or in relapsing MS patients compared with healthy donors (HDs) or patients affected by other non-inflammatory neurological disorders (7). This overproduction of EVs in CSF is mirrored in higher numbers of EVs released by platelets, leukocytes, and monocytes in the blood of MS patients compared to HC (8).

The activation of CD4+ autoreactive T cells and their differentiation into a Th1 or Th17 phenotype are crucial events in the initial steps of MS, though, in some forms of MS, the monocytes are thought to be the primary cell type responsible for cellular pathology and tissue damage (9–13). In MS pathology, activated monocytes largely represent the inflammatory infiltrate, depending on the type of disease and stage of demyelinating activity. Despite the considerable presence of activated monocytes in MS lesions, the features of circulating monocytes are little understood, since very few studies focus on their phenotypes in MS patients. Blood monocytes are thought to play a key role due to their capability to secrete various immunoregulatory cytokines that regulate crucial immune functions (14). The M1/M2 paradigm is currently used to categorize the monocyte/macrophage functions (15, 16). Generally, M1 indicates the classically activated macrophages and M2 refers to the alternatively activated macrophages (17, 18). The polarization of macrophages is expressed in terms of cell surface receptor expression, effect or functions, cytokine, and chemokine production (19). For example, interleukin-1β (IL-1B), IL-18, and TNFalpha (TNFa) are typically associated with M1 activation, whereas arginase 1 (Arg1), chitinase 3 like 1 (CHI3L1), and IL-10 fit an M2 signature (20).

Disease modifying drugs (DMDs) currently used for MS, such as recombinant interferon-beta (IFNb)-1a or -1b and glatiramer acetate (GA), have been mostly designed to target the activation and function of pathogenic auto-aggressive T cells in MS, but the effects on both monocyte or macrophage function have been clearly reported (21–23). Interestingly, in a mouse model, GA has been reported to promote development of anti-inflammatory type II monocytes (24). More recently, we have demonstrated (25) the synergistic effects of GA and Minocycline on peripheral blood monocyte-derived dendritic cells in MS patients.

Recently, the involvement of ATP and its related receptors in the phlogistic process has been recognized (26). In particular, the purinergic P2X7 receptor (P2X7R), especially expressed on cells of hematopoietic origin (26), seems to play a crucial role in macrophage/microglial function as it is able to regulate cytokine production and apoptosis. Furthermore, as evidence indicates the P2X7R upregulation during inflammation, antagonists of this receptor could be useful novel anti-inflammatory drugs. The presence of ATP in the extracellular milieu is thought to trigger signals that lead APCs to promote the innate immune response (27). Notably, a lot of cytokines produced by P2X7R activation [e.g., IL-1B, interleukin-18 (IL-18), interleukin-6 (IL-6) and TNFa] may promote innate immunity. We have previously demonstrated that P2X7R, IL-1B, and CD39 expressed in monocytes from both MS and healthy controls can be affected by GA (28). Bianco et al. have described a microparticle (MP) shedding from microglia cell surface induced by stimulation of P2X7R by ATP or the selective agonist benzoyl-ATP (BzATP). They also showed that MPs store and release the inflammatory cytokine IL-1B (29).

Stimulation of P2X7R triggers the rapid assembly of inflammasome signaling complexes (30) that include the NLR family pyrin domain containing 3 (NLRP3), PYD and CARD domain-containing protein (PYCARD), and Caspase-1, the protease responsible for the IL-1B conversion from an inactive precursor to an active secreted cytokine. In recent years, inflammasomes have been gaining increasing attention in MS and its animal model, experimental autoimmune encephalomyelitis (EAE). As a matter of fact, NLRP3-deficient mice show an improvement in EAE reducing Th1 and Th17 cells in the peripheral lymphoid tissues and spinal cord (31, 32). Moreover, IFNb interferes with IL-1β production in mouse bone marrow-derived macrophages by inhibiting NLRP1 and NLRP3 inflammasome activity and by inducing IL-10 (33).

The aim of this study was to investigate the release of MVs in P2X7R-stimulated monocytes from MS patients and HDs and to see how current immunomodulatory treatments may affect such production. We also aimed to assess the polarization of monocytes in M1 and M2 subtypes. The treatment effect was also evaluated on the inflammasome components.

## Materials and Methods

### Patients and Controls

Twenty relapsing–remitting DMDs untreated MS patients were selected from the MS Center, Neurology Unit, Department of Medical and Surgical Sciences, University of Foggia. The male to female ratio was 1/4; the mean age at the time of blood withdrawal was 35 ± 9 years, the mean EDSS score was 2.9 ± 1.2, the mean disease duration 2.4 ± 1.3. All the patients were clinically inactive at the time of blood withdrawal, therefore totally steroid free. Twenty HDs (8 M/12 F, mean age 34 ± 9) were also included into the study. Venous blood samples (20 ml each) were obtained from all subjects in order to obtain purified monocytes from total peripheral blood mononuclear cells (PBMNCs), before and then during treatment at different time points, 2, 6, and 12 months respectively. A subgroup of nine MS patients was given IFNb treatment (44 μg/3 times a week subcutaneously) and a subgroup of five was given teriflunomide (14 mg/once daily orally). All the patients were responsive to treatments since neither relapses nor steroid courses were reported during the period of the study. Another group of six MS patients consuming fingolimod, after switching from a first-line therapy, was included into the study and evaluated only at 12 months of treatment.

### Ethics Statement

The study has been reviewed and approved by the Ethical Committee of the Ospedali Riuniti Foggia/University of Foggia. Patients and HDs were given an informed consent to sign before blood samples were taken.

### Preparation of Monocytes

Peripheral blood mononuclear cells from untreated patients with MS and HDs were isolated from freshly drawn venous blood by density centrifugation, using a Lymphosep^®^, lymphocyte separation media (*d* = 1.077 g/ml) gradient (Biowest) as described by the manufacturer.

10 µl of the cell suspension was mixed with 10 µl of 4% trypan blue solution and live/dead cells were counted in a Bio-Rad TC-10 Automated Cell Counter in order to plate 1 × 10^6^ cells into 35 mm dishes and they were then incubated at 37°C and 5% CO_2_ for 2 h, the time required to let the monocytes adhere to the plate.

### MV Isolation and Quantification

Monocyte-derived MVs were isolated by Differential Centrifugation following the method described by Bianco et al. (29). Briefly, cultured monocytes were washed with PBS and then labeled with the fluorophore-conjugated phosphocholine compound NBD C_6_-Sphingomyelin dye (5 µM) for 5 min. After extensive washing, monocytes were stimulated with BzATP (200 µM for 30 min) dissolved in Krebs-Ringer solution (KRH, 125 mM NaCl, 5 mM KCl, 1.2 mM MgSO_4_, 1.2 mM KH_2_PO, 2 mM CaCl_2_, 6 mM d-glucose, and 25 mM HEPES/NaOH, pH 7.4) or unstimulated using KRH buffer without BzATP as control. Supernatants containing shed MVs were collected and centrifuged at 300 × *g* for 10 min at 4°C two times in order to remove cells and debris. Finally, the total green fluorescence of shed vesicles present in the 200 µl of supernatant was assayed at 485/535 nm with a spectrophotometric system (1420 Multilabel Counter Victor 2; Wallac). Arbitrary fluorescence units (AU) of BzATP-stimulated monocytes were compared with arbitrary fluorescence units of unstimulated monocytes.

### RNA Purification and Quantitative Real-time PCR (qPCR)

Total RNA from monocytes was isolated using the PureLink^®^ RNA Mini Kit (Ambion), according to the manufacturer’s instructions. On-column digestion of DNA during RNA purification was also performed using PureLink ™DNase to exclude possible genomic contamination. The concentration of RNA was determined by measuring the absorbance at 260 nm using a NanoDrop 1000 Spectrophotometer. RNA (200 ng) was reverse transcribed into cDNA (High Capacity cDNA Reverse Trascription Kits—Applied Biosystem) that was used for subsequent PCR amplification using TaqMan^®^ Universal Master MixII (Applied Biosystems) and gene specific Taqman^®^ assays (Applied Biosystems) for P2X7R (Hs00175721_m1), IL-1B (Hs00174097_m1), TNFa (Hs01113624_g1), IL-6 (Hs00985639_m1), IL-18 (Hs01038788_m1), Arg1 (Hs00968979_m1), CHI3L1 (Hs00171080_m1), interleukin-10 (IL-10) (00961622_m1), NLRP3 (Hs00918082_m1), PYCARD (Hs01547324_gH), and CASP1 (Hs00354836_m1). qPCR was performed with a Step One real-time PCR system (Applied Biosystems). The relative mRNA levels were calculated using the comparative Ct method, using beta-actin (Hs01060665_g1) as endogenous control.

### Statistical Analysis

Statistical comparison between fluorescence of MVs, P2X7R, IL-1B, IL-6, IL-18, TNFa, Arg1, CHI3L1, IL-10, NLRP3, PYCARD, CASP1 between differently *in vitro* stimulated monocytes was performed using the non-parametric Mann–Whitney *U* test for two independent samples or the multiple comparison non-parametric Kruskal–Wallis test for independent samples followed by a *post hoc* test. Longitudinal comparison between fluorescence of MVs and also P2X7R, M1 and M2 markers as well as inflammasome components on monocytes from MS patients before and after 2, 6 or 12 months of IFNb or teriflunomide treatment was performed using the multiple comparison Friedman non-parametric test followed by the non-parametric Wilcoxon test for two related samples. *p*-Values < 0.05 were considered significant.

## Results

### IFNb and Teriflunomide Affect Monocyte-Derived MV Production

**Figure 1:**
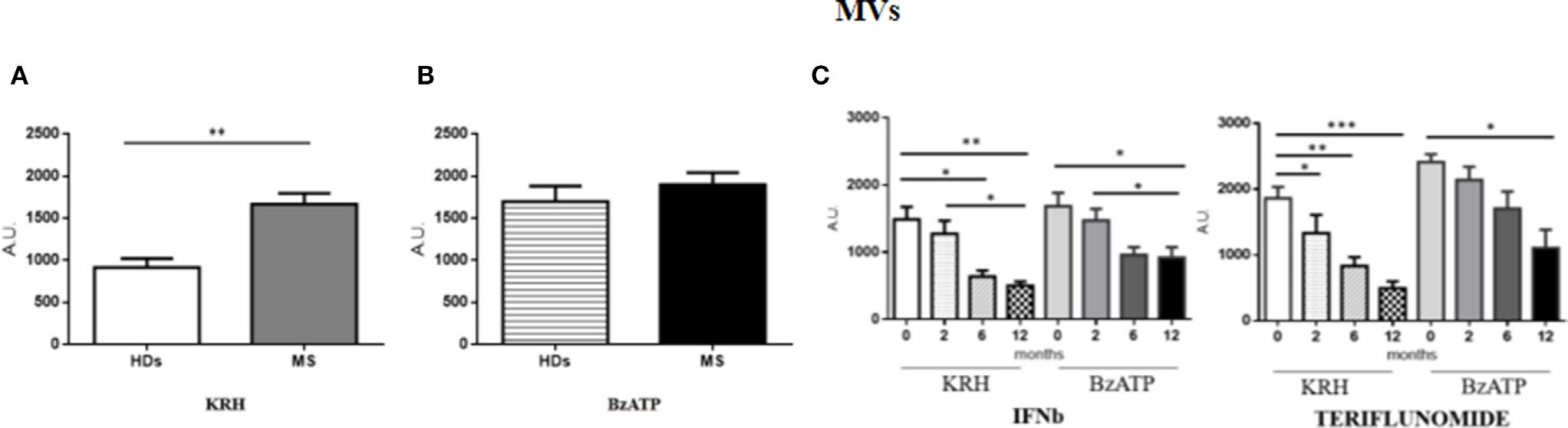
Comparison of microvesicle (MV) production between untreated MS and healthy donors (HDs): **(A)**. monocytes-derived MVs released by unstimulated cells Krebs-Ringer solution (KRH); **(B)** MVs produced by monocytes challenged with benzoyl-ATP (BzATP). Data obtained by spectrophotometric analysis are expressed as mean ± SEM of fluorescence arbritary unit (A.U.); **(C)** effect of interferon-beta (IFNb) and teriflunomide treatments on monocyte-derived microvescicles in MS patients followed from the baseline up to 12 months of therapy.

The amount of MVs was only found to be higher (*p* < 0.01) in MS patients compared to HD in unstimulated monocytes, while, upon stimulation with BzATP, only the HD monocytes exhibited a significant increase in fluorescence (*p* < 0.001). Both IFNb and teriflunomide were able to reduce the MV production, but with teriflunomide the effect was even more evident. As far as IFNb is concerned, a significant reduction of MV release (*p* < 0.05) was evident starting from the sixth month of treatment and it further decreased after 1 year of therapy (*p* < 0.01), whereas teriflunomide treated patients already showed a progressive reduction from the second month after the drug administration (*p* < 0.05), more significant after 6 months (*p* < 0.01) and even more after 1 year of treatment (*p* < 0.001) (see Figures 1A–C).

### Teriflunomide but Not IFNb Is Associated with a Downregulation of P2X7R

**Figure 2:**
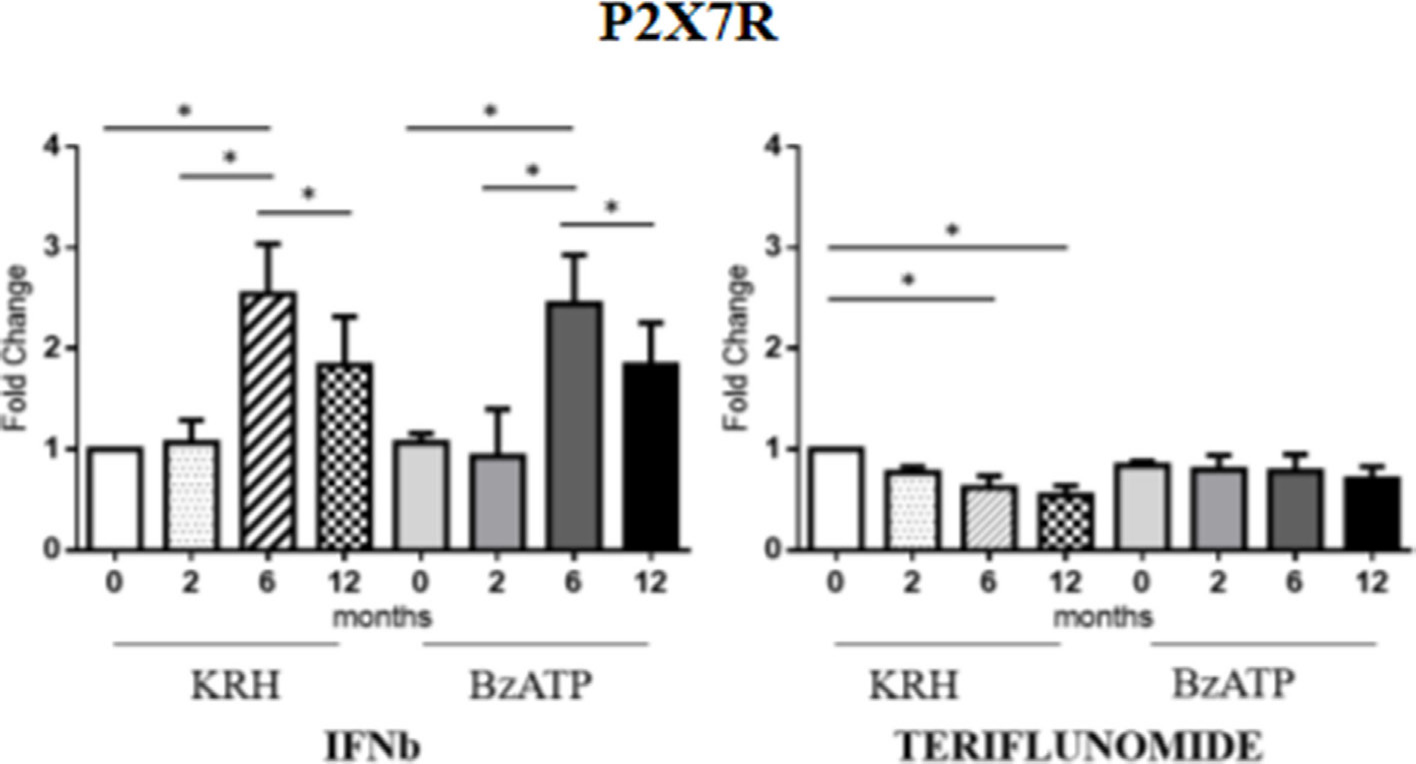
Purinergic P2X7 receptor (P2X7R) expression level by RT-PCR in unstimulated Krebs-Ringer solution (KRH) and benzoyl-ATP (BzATP)-stimulated monocytes in MS patients from the baseline to 12 months of interferon-beta (IFNb) and teriflunomide treatments. Data are expressed as mean ± SEM of fold change values (*p < 0.05; **p < 0.01; ***p < 0.001).

No actual differences in P2X7R expression between MS patients and HDs were found. Longitudinal analysis showed the different effects of the two drugs. In the case of IFNb, unstimulated monocytes displayed an increase in gene expression of the receptor up to 6 months that was then reduced after 12 months (*p* < 0.05) of treatment reaching the value of the pretreatment. The same was observed in BzATP-stimulated monocytes. However, teriflunomide in unstimulated monocytes already downregulated the receptor expression after 6 months of treatment (*p* < 0.05) also keeping the expression low after 12 months. In BzATP-stimulated monocytes, however, there were no statistically significant differences, as reported in Figure 2.

### Teriflunomide Is More Effective than IFNb in Downregulating M1 Markers

**Figure 3:**
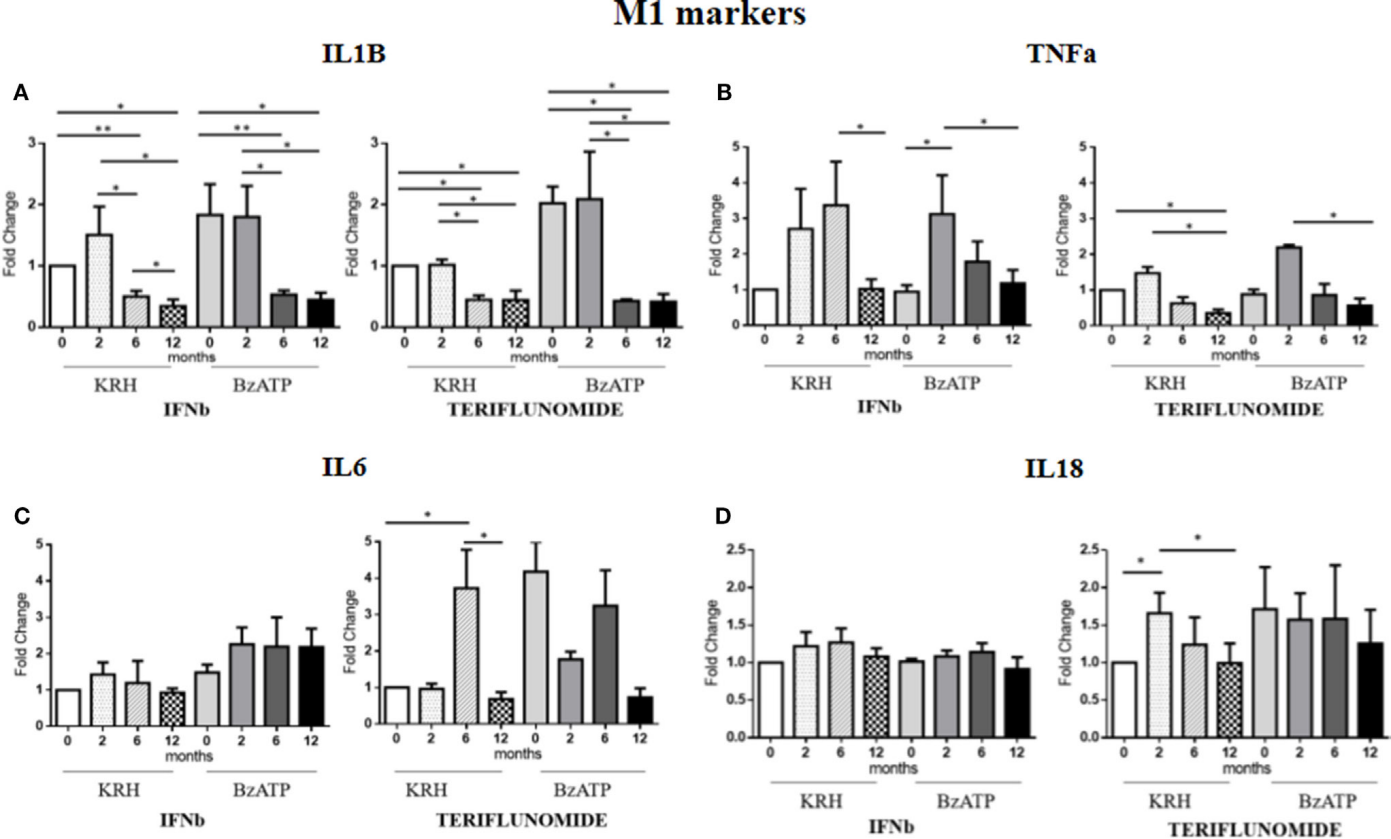
Expression level of M1 markers evaluated by RT-PCR: interleukin-1β (IL-1B) **(A)**, TNFalpha (TNFa) **(B)**, IL-6 **(C)**, and IL-18 **(D)** in unstimulated Krebs-Ringer solution (KRH) or benzoyl-ATP (BzATP)-stimulated monocytes in MS patients at baseline and after 2, 6, and 12 months of treatments with interferon-beta (IFNb) and teriflunomide. Data are expressed as mean ± SEM of fold change values (*p < 0.05; **p < 0.01; ***p < 0.001).

Comparing the untreated MS with the HDs, no significant differences were evident for IL-1B gene expression. Only the stimulation with BzATP was able to increase (*p* < 0.05) the IL-1B expression, and this was even clearer in the control group. Both treatments in either unstimulated or stimulated conditions downregulated IL-1B expression starting from 6 months of therapy (*p* < 0.05), as reported in Figure 3A.

The expression of the proinflammatory cytokine TNFa showed no significant differences between the MS and the HDs. From the longitudinal analysis, two different effects of the two drugs emerged. In the case of IFNb treatment, in both unstimulated and stimulated conditions, there was a substantial increase in cytokine gene expression up to 6 months of treatment that was reduced at 12 months (*p* < 0.05) of therapy, again reaching the pretreatment values. In contrast, teriflunomide, after a non-significant increase in the expression at 2 months of treatment, induced a downregulated TNFa expression at 6 months of treatment that became statistically significant at 12 months (*p* < 0.05), in both unstimulated and BzATP-stimulated monocytes (Figure 3B).

In the case of IL-6, the gene expression in MS patients was similar to that in the HDs. After conditioning with BzATP, a clearly significant increase in IL-6 expression was detected in both the control group (*p* < 0.01) and in untreated MS patients (*p* < 0.001). IFNb therapy did not modify IL-6 expression, while teriflunomide seemed to exert its inhibitory effect at 12 months (*p* < 0.05) of therapy, markedly attenuating the increase that was recorded at 6 months (*p* < 0.05). This was statistically significant in unstimulated monocytes (Figure 3C). Concerning IL-18, also in this case no significant difference between the MS and HD group was evident. Only upon stimulation of monocytes with BzATP was a significant increase in IL-18 expression observed in both control group and untreated MS patients (*p* < 0.05). IFNb therapy did not change the expression of IL-18, while in the case of teriflunomide, we observed a statistically significant decrease between the second and 12th month of treatment (*p* < 0.05). The statistical significance was only observed in unstimulated monocytes, as shown in Figure 3D.

### IFNb and Teriflunomide Mostly Modulate M2 Markers

**Figure 4:**
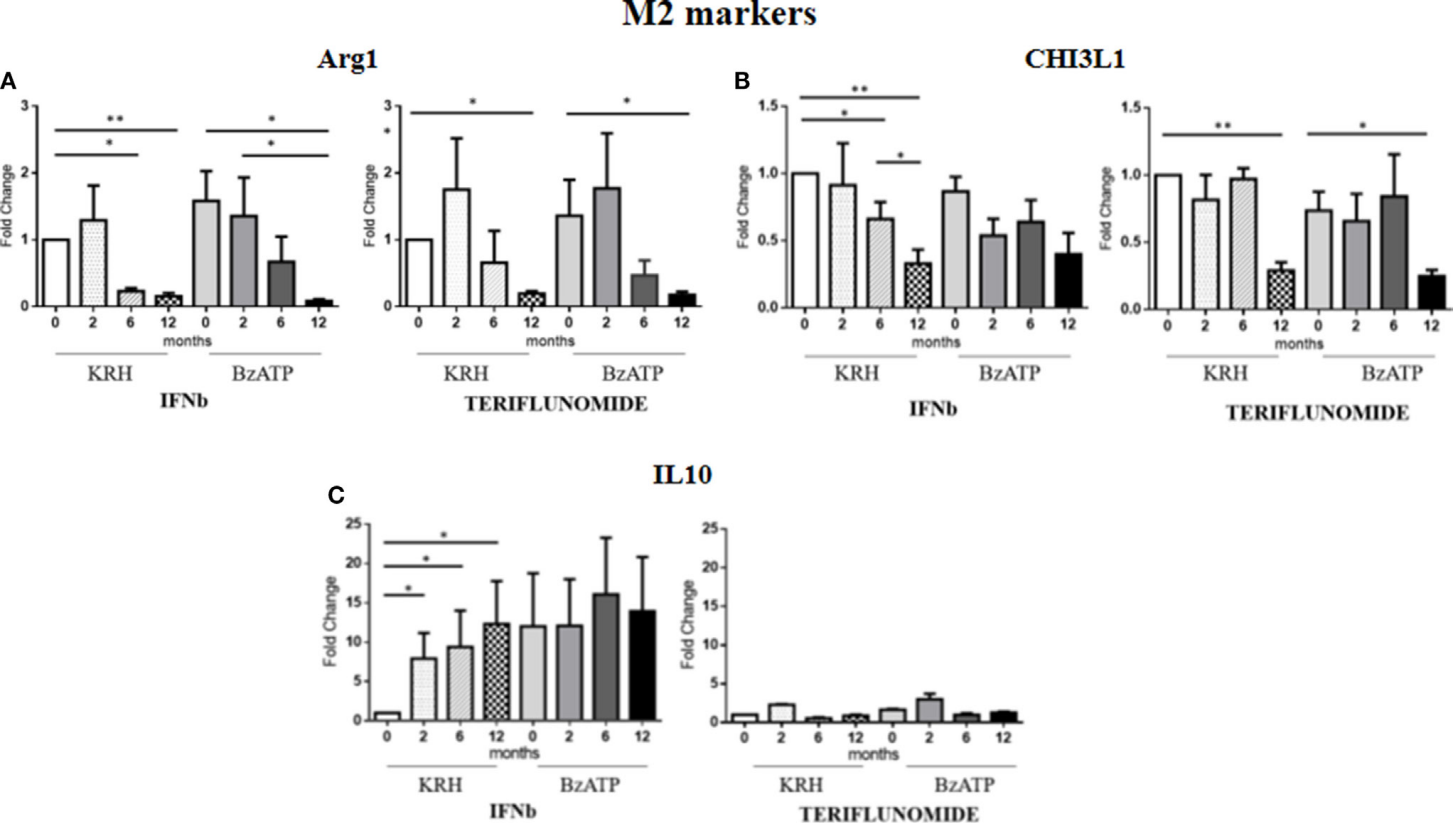
Expression level of M2 markers measured by RT-PCR: arginase 1 (Arg1) **(A)**, chitinase 3 like 1 (CHI3L1) **(B)** and IL-10 **(C)** in monocytes with or without benzoyl-ATP (BzATP) stimulation in MS patients monitored during 12 months of interferon-beta (IFNb) and teriflunomide administration (*p < 0.05; **p < 0.01; ***p < 0.001).

For Arg1, the comparison between the untreated MS group and the control group revealed a clearly higher expression in MS patients. This occurred both in BzATP in unstimulated and BzATP-stimulated monocytes (*p* < 0.05). Both treatments, before and after addition of BzATP, were able to significantly decrease the expression in a time-dependent manner since this reduction was mostly evident after 1 year of treatment (*p* < 0.01), as shown in Figure 4A.

The CHI3L1 gene expression was higher in MS patients than in HDs, both before (*p* < 0.01) and after stimulation with BzATP (*p* < 0.01). Following the therapy with IFNb, we observed a gradual downregulation starting from 6 months of treatment (*p* < 0.05), which was more evident after 12 months (*p* < 0.01). This effect was only statistically significant in the unstimulated conditions. In patients treated with teriflunomide, CHI3L1 gene expression reduction was only evident at 12 months of treatment (*p* < 0.01), in both unstimulated and stimulated conditions (Figure 4B).

No significant differences between patients and controls emerged from the analysis of IL-10 expression. However, it is interesting to note that in non-stimulus conditions only IFNb was able to induce an increase (*p* < 0.05) in this anti-inflammatory cytokine, 10 times higher than the baseline. In contrast, teriflunomide did not alter in any way the expression of this gene as can be seen in the graphs in Figure 4C.

### Teriflunomide Is More Effective than IFNb in Downregulating Inflammasome Components

**Figure 5:**
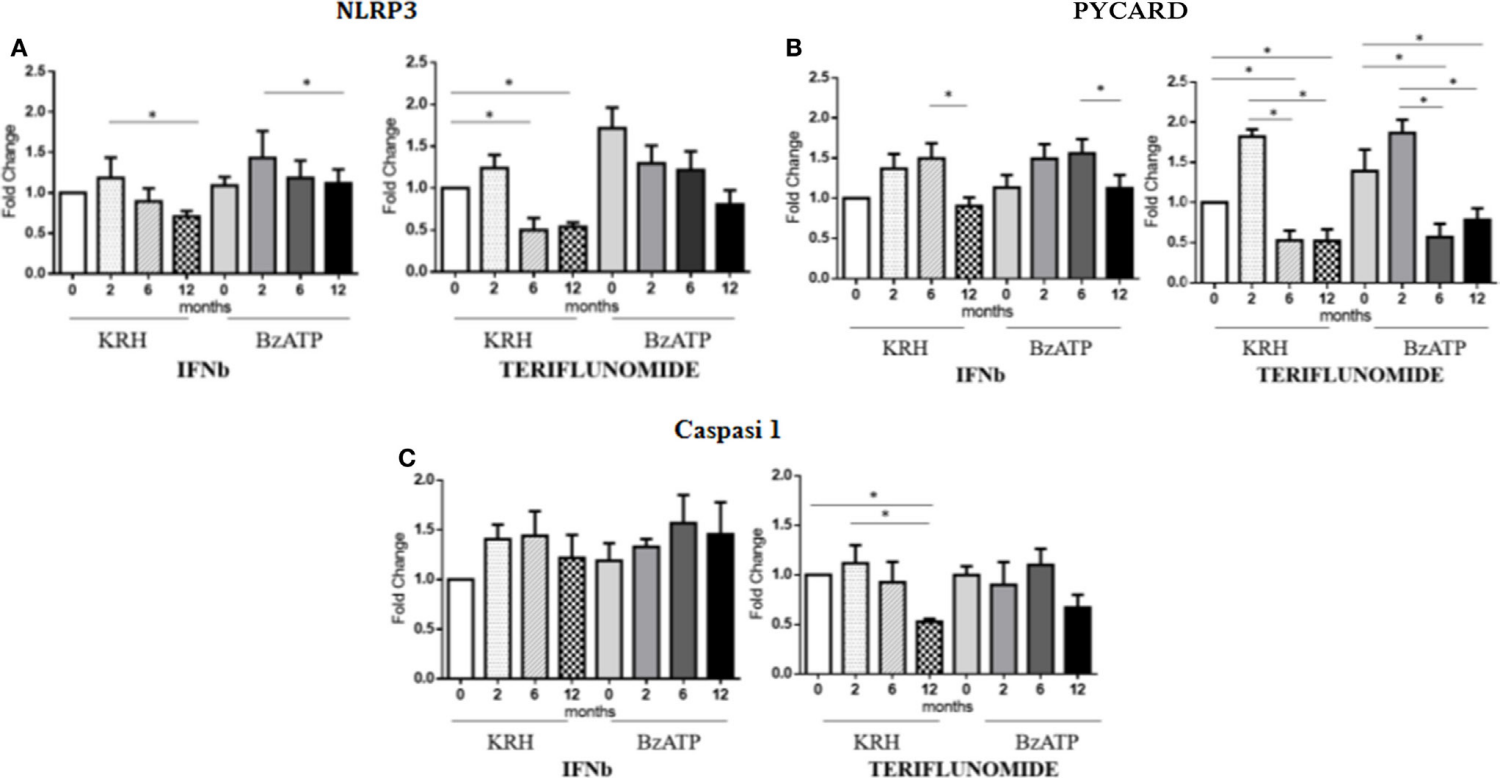
mRNAexpression level of inflammasome components NLR family pyrin domain containing 3 (NLRP3) **(A)**, PYD and CARD domain-containing protein (PYCARD) **(B)** and Capasi-1 **(C)** in either unstimulated or benzoyl-ATP (BzATP)-stimulated monocytes in MS patients observed during 12 months of interferon-beta (IFNb) and teriflunomide treatments (*p < 0.05; **p < 0.01; ***p < 0.001).

The stimulation with BzATP was able to increase (*p* < 0.05) the expression of NLRP3 and this was more evident in the control group. IFNb therapy only reduced the expression of NLRP3 at 12 months of treatment (*p* < 0.05), in both unstimulated and stimulated conditions, while for teriflunomide a downregulatory effect was already evident starting from the sixth month of dosing (*p* < 0.05) but this was only statistically significant in unstimulated monocytes, as shown in Figure 5A. Interestingly, only teriflunomide clearly reduced PYCARD expression (in both unstimulated and stimulated conditions) and Caspase-1 expression (only in unstimulated conditions) especially at 12 months of treatment (*p* < 0.05) (see Figures 5B,C).

### Effects of Treatments at 12 months of Therapy: Comparison between IFNb, Teriflunomide and Fingolimod

**Figure 6:**
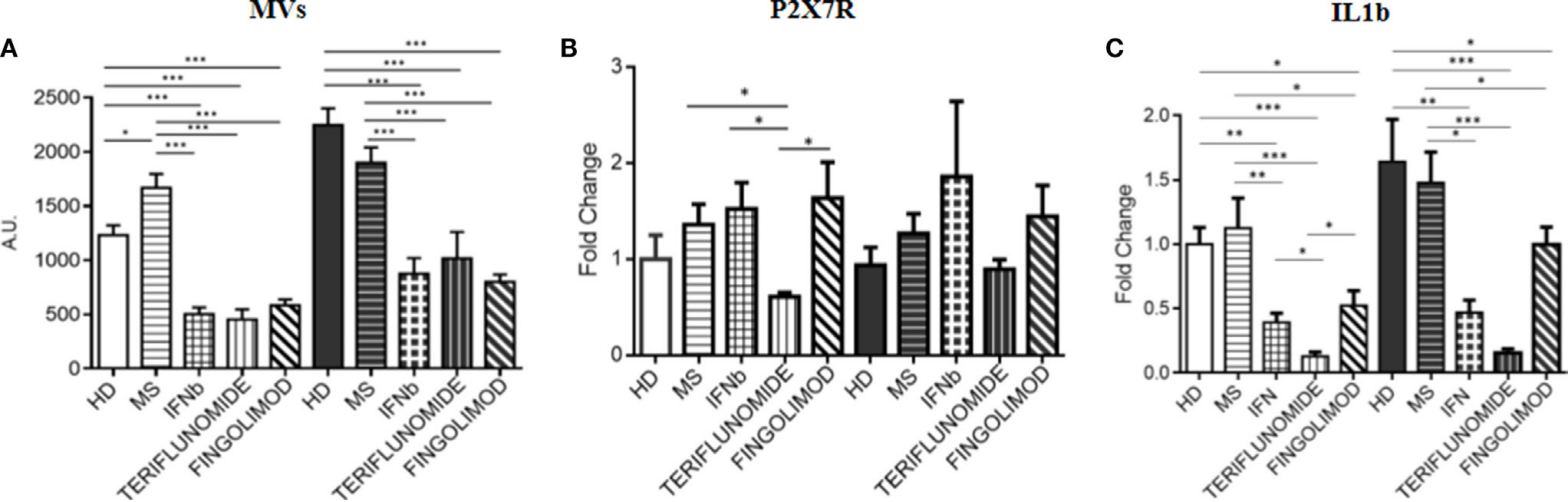
Effects of different treatments at 12 months of therapy on MV production **(A)**, on purinergic P2X7 receptor (P2X7R) **(B)**, and interleukin-1β (IL-1B) **(C)** expression: comparison between interferon-beta (IFNb), teriflunomide, and fingolimod (*p < 0.05; **p < 0.01; ***p < 0.001).

Since fingolimod is a second-line treatment and, the treated patients always switched from a first-line therapy, a pretreatment point was not available for patients receiving this drug. Therefore, in order to assess the effects of the three treatments, comparisons after 12 months of treatment were performed. All data were normalized to unstimulated HD monocytes. The analysis of the data showed that all treatments were able to significantly reduce MV production compared to MS patients (*p* < 0.001), and also compared to HDs (*p* < 0.001). This effect was observed in both unstimulated and stimulated conditions. No difference was observed between the three therapies (Figure 6A). It is worth considering that the expression of the P2X7 receptor, only in the case of teriflunomide 12 months drug administration, showed a downregulatory effect in unstimulated monocytes, as shown in Figure 6B. All three drugs, however, reduced IL-1B expression compared with the control group and with the untreated patients, and this was evident in both unstimulated and stimulated conditions. In particular, the decrease was more evident in the case of teriflunomide (*p* < 0.001) (Figure 6C).

Only teriflunomide decreased M1 markers, such as TNFa and IL-6, whereas both first- and second-line drugs were able to reduce Arg1 and CHI3L1 gene expression (results not shown). To investigate a possible mechanism of action through which the drugs reduced MV release, we also analyzed the expression of inflammasome components. Notably, NLRP3 was reduced by IFNb and teriflunomide, while PYCARD and Caspase-1 were only reduced by teriflunomide (results not shown).

## Discussion

In this paper, we have demonstrated for the first time that the drugs currently used for the treatment of MS seem to interfere with the production of MVs from monocytes in MS patients.

We selected IFNb and teriflunomide as first-line drugs because we wanted to evaluate the effects of two drugs with potentially different mechanisms of action, immunomodulatory for IFNb and more immunosuppressive for teriflunomide.

The aim of studying monocytes either in unstimulated or BzATP-stimulated conditions was to report the specific effects of treatments on MV release in conditions mimicking both a more inactive or a more active phase of the disease. Even considering the evident limitations of this kind of observation *in vitro*, the treatment effects were mostly evident in BzATP-stimulated conditions.

From the analysis of our data, it is evident that the mean fluorescence value, which indicates the amount of MV produced, is only higher in MS patients than in HD in unstimulated monocytes, while, upon stimulation with BzATP, only the HD monocytes exhibited a significant increase in fluorescence, as if the monocytes of MS patients were already *per se* stimulated and further stimulus did not modify their status.

It is worth reporting that both treatments decreased MV production and in a time-dependent manner. Therefore, the longer the patients were treated, the greater was the decrease. IFNb started showing this effect after 6 months of treatment, while teriflunomide displayed a more prompt inhibition. Our data are consistent with the results published by Sheremata et al. (34), which showed a marked reduction in plasma levels of microparticles produced by CD31+ endothelial cells in patients with MS starting from 26 weeks of IFNb treatment. Jimenez et al. (35) had previously observed the capability of IFNb to inhibit *in vitro* the formation of MVs produced by endothelial cells exposed to the serum of MS patients. As far as the teriflunomide is concerned, the drug has only recently been approved and made commercially available. Little is known about its mechanism of action and there are no studies on its effects on MVs. We know that teriflunomide is the active metabolite of leflunomide, an immunosuppressive drug with anti-inflammatory properties approved for rheumatoid arthritis. The mechanism of action of teriflunomide seems to be related to inhibition of dihydroorotate dehydrogenase, a key mitochondrial enzyme for the *de novo* synthesis of pyrimidine, which is essential for the proliferation of B and T cells. Therefore, its mechanism of action may be considered as immunosuppressive. The effects on monocytes are not known yet. We showed for the first time that this drug is able to modulate the MV production in monocytes and this is evident from the second month of treatment.

All treatments examined were capable of reducing the production of IL-1B, but this decrease was associated with downregulation of the P2X7R only in the case of teriflunomide. This was statistically significant only in unstimulated monocytes, may be due to the small data set recruited in this study. Moreover an alternative pathway for the MV release which is independent of P2X7 receptor activation may be hypothesized as recently it has been emerging.

However, since the activation of the P2X7 receptor triggers the inflammasome assembly which may induce the release of IL-1B through the MVs, we decided to assess the expression levels of the inflammasome components, such as NLRP3, PYCARD and Caspase-1. IFNb therapy reduced the expression of NLRP3 only after 12 months of treatment, in both unstimulated and stimulated conditions, while in the case of teriflunomide the downregulatory effect was already evident at 6 months and even more evident after 12 months of treatment. Interestingly, only teriflunomide reduced PYCARD and Caspase-1 expression in both conditions, especially at 12 months of treatment. It is worth considering that IFNb seems to exert its ability to inhibit the release of IL-1B *via* two routes: the downregulation of the gene coding for the protein of the inflammasome complex and the induction of IL-10 which is known to prevent the conversion of pro-active IL-1B into IL-1B.

TNFalpha M1 marker was downregulated by both treatments even if the effect was delayed, after 12 months for IFNb and 6 months for teriflunomide. There were no IFNb effects on IL-6 or IL-18, whereas teriflunomide showed a downregulating effect at 12 months of treatment.

Alternately activated monocytes seem to prevail in the disease, given the increase in gene expression of Arg1 and CHI3L1 found in untreated MS patients compared with healthy controls. The treatments, however, were able to reduce this overexpression. Our data confirm in humans the results published by Xu et al. (36) in a preclinical setting. Using a high throughput microarray gene technology that can detect up to 22,000 genes, they found an overexpression of the Arg1 gene and that its inhibition improved the course of EAE in the spinal cord of EAE mice, the animal model for MS. Arg1 is an enzyme that converts arginine, competing with the nitric oxide (NO) synthase 2 enzyme involved in the production of NO that was previously considered cytotoxic and demyelinating, even though recent studies highlight a protective role for NO. Therefore, according to our data, an increased expression of Arg1 would unbalance the reaction toward the production of ornithine and urea rather than protective NO. But, this is only a hypothesis in which Arg1 could have an independent pathogenetic role from NO, therefore it needs closer examination. Furthermore, according to the authors, the inhibition of Arg1 with the synthetic antagonist ABH improved the course of EAE. We found a reduced expression of the gene Arg1 as a result of drug treatment of MS patients. Furthermore, this was also evident for CHI3L1, a glycoprotein secreted by activated macrophages and upregulated in many inflammatory conditions, such as cancer, cardiovascular diseases, and neurodegenerative diseases. A possible role for CHI3L1 in the pathogenesis of MS is emerging. Moreover, Malmeström et al. report that the CSF of MS patients has elevated levels of CHI3L1, higher than those of healthy controls that decreased following treatment with mitoxantrone and natalizumab (37). In line with these data, we found a higher gene expression of this glycoprotein in the monocytes of untreated MS patients and a marked decrease in response to therapy. Nevertheless, if we consider Arg1 and CHI3L1 as markers of protective innate immunity, it is difficult to understand why it is inhibited by potentially beneficial treatments. As already suggested by others (38, 39), we hypothesize a possible role of alternately activated monocytes already in the early stages of the disease. Therefore, at the beginning of the disease, all the inflammatory components are activated, including the M2 component, which is considered to be anti-inflammatory that could balance the proinflammatory one represented by M1 monocytes. In the course of treatment, as a consequence of the reduced expression of proinflammatory cytokines of the M1 component, we observed a downregulation of the M2 markers. The content of the MVs was not the aim of the present study, nevertheless Garzetti et al. (40) recently reported that activated macrophages release MVs containing polarized M1 and M2 mRNAs so to assume that the polarization of the cell reflects the polarization of MVs released. Therefore, we can hypothesize that the material carried by the MVs (e.g., cytokines, miRNAs, RNAs, membrane receptors, etc.) is able to modulate inflammation also by affecting the phenotype of monocytes.

Finally, we compared the treatment effects, including fingolimod, at 12 months, since fingolimod, as a switching second-line therapy, could not have had a baseline timepoint. Fingolimod was as effective as IFNb and teriflunomide in reducing the MV production as well as IL-1B expression, while no effect was reported on P2X7R, the other M1, M2 markers or the inflammasome components. This final comparison confirms the more extensive effect of teriflunomide.

## Conclusion

Despite the increasing recognized relevance of EVs, the information on the role of MVs in MS is still incomplete. Current literature suggests that MV detection may represent a very promising strategy to gain pathogenic information, since they have been indicated as important mediators of intracellular communication, they are emerging as new biomarkers of tissue damage and they could be especially helpful to examine inaccessible districts. However, to date, the role of monocytes-derived MVs in Multiple Sclerosis is still very little known and remains to be investigated. Our results show for the first time that the drugs currently used for the treatment of MS seem to interfere with the production of MVs from monocytes in MS patients. The small number of patients certainly represents a limitation for drawing clear conclusions from this study, which need to be confirmed in a wider population. However, while leaving some questions unresolved, the present observations suggest new molecular targets for drugs currently used in MS, providing useful evidence leading to the identification of additional and possibly more effective approaches to be addressed in the near future.

## Ethics Statement

The study has been reviewed and approved by the Ethical Committee of the Ospedali Riuniti Foggia/University of Foggia. Patients and HDs were given an informed consent to sign before blood samples were taken.

## Author Contributions

MB and AA performed the experiments; RG and VF selected the patients; MB analyzed the data and wrote the manuscript; CA supervised the project, procured funds, supervised, and corrected the manuscript.

## Conflict of Interest Statement

The authors declare that the research was conducted in the absence of any commercial or financial relationships that could be construed as a potential conflict of interest.

## Abbreviations

MS, multiple sclerosis; CNS, central nervous system; EVs, extracellular vesicles; Abs, apoptotic bodies; MVs, microvesicles; EXOs, exosomes; CSF, human cerebrospinal fluid; CIS, clinical isolated syndrome; HDs, healthy donors; IL-1B, interleukin-1β; IL-18, interleukin-18; TNFalpha, TNFa tumor necrosis factor-α; Arg1, arginase 1; CHI3L1, chitinase 3 like 1; IL-10, interleukin-10; IL-6, interleukin-6; DMDs, disease modifying drugs; IFNb, interferon-beta; GA, glatiramer acetate; P2X7R, purinergic P2X7 receptor; BzATP, benzoyl-ATP; MP, microparticle; NLRP3, NLR family pyrin domain containing 3; PYCARD, PYD and CARD domain-containing protein; PBMNCs, peripheral blood mononuclear cells; KRH, Krebs-Ringer solution; NOS2, nitric oxide synthase 2; NO, nitric oxide.

## References

[1] Sospedra M Martin R. Immunology of multiple sclerosis. Annu Rev Immunol (2005) 23:683–747. 10.1146/annurev.immunol.23.021704.115707 15771584

[2] Raposo G Stoorvogel W. Extracellular vesicles: exosomes, microvesicles, and friends. J Cell Biol (2013) 200(4):373–83. 10.1083/jcb.201211138 23420871 PMC3575529

[3] Crescitelli R Lässer C Szabó TG Kittel A Eldh M Dianzani I Distinct RNA profiles in subpopulations of extracellular vesicles: apoptotic bodies, microvesicles and exosomes. J Extracell Vesicles (2013) 2:20677. 10.3402/jev.v2i0.20677 24223256 PMC3823106

[4] Frühbeis C Fröhlich D Kuo WP Krämer-Albers EM Extracellular vesicles as mediators of neuron-glia communication. Front Cell Neurosci (2013) 7:182 10.3389/fncel.2013.00182 24194697 PMC3812991

[5] Lai CP Breakefield XO. Role of exosomes/microvesicles in the nervous system and use in emerging therapies. Front Physiol (2012) 3:228. 10.3389/fphys.2012.00228 22754538 PMC3384085

[6] Sáenz-Cuesta M Osorio-Querejeta I Otaegui D. Extracellular vesicles in multiple sclerosis: what are they telling us? Front Cell Neurosci (2014) 8:100. 10.3389/fncel.2014.00100 24734004 PMC3975116

[7] Verderio C Muzio L Turola E Bergami A Novellino L Ruffini F Myeloid microvesicles are a marker and therapeutic target for neuroinflammation. Ann Neurol (2012) 72(4):610–24. 10.1002/ana.23627 23109155

[8] Sáenz-Cuesta M Irizar H Castillo-Triviño T Muñoz-Culla M Osorio-Querejeta I Prada A Circulating microparticles reflect treatment effects and clinical status in multiple sclerosis. Biomark Med (2014) 8(5):653–61. 10.2217/bmm.14.9 25123034

[9] Davidson A Diamond B Autoimmune diseases. N Engl J Med (2001) 345:340–50. 10.1056/NEJM200108023450506 11484692

[10] Raine CS. The Dale E. McFarlin memorial lecture: the immunology of the multiple sclerosis lesion. Ann Neurol (1994) 36(Suppl):S61–72. 10.1002/ana.410360716 8017891

[11] Lucchinetti C Bruck W Parisi J Scheithauer B Rodriguez M Lassmann H. Heterogeneity of multiplesclerosis lesions: implications for the pathogenesis of demyelination. Ann Neurol (2000) 47:707–17. 10.1002/1531-8249(200006)47:6<707::AID-ANA3>3.0.CO;2-Q 10852536

[12] Steinman L Multiple sclerosis: a coordinated immunological attack against myelin in the central nervous system. Cell (1996) 85:299–302. 10.1016/S0092-8674(00)81107-1 8616884

[13] Noseworthy JH Lucchinetti C Rodriguez M Weinshenker BG Multiple sclerosis. N Engl J Med (2000) 343:938–52. 10.1056/NEJM200009283431307 11006371

[14] Kouwenhoven M Teleshova N Ozenci V Press R Link H Monocytes in multiple sclerosis: phenotype and cytokine profile. J Neuroimmunol (2001) 112(1–2):197–205. 10.1016/S0165-5728(00)00396-9 11108949

[15] Biswas SK Mantovani A. Macrophage plasticity and interaction with lymphocyte subsets: cancer as a paradigm. Nat Immunol (2010) 11:889–96. 10.1038/ni.1937 20856220

[16] Italiani P Boraschi D From monocytes to M1/M2 macrophages: phenotypical vs functional differentiation. Front Immunol (2013) 5:514 10.3389/fimmu.2014.00514 PMC4201108 25368618

[17] Mantovani A Sozzani S Locati M Allavena P Sica A. Macrophage polarization: tumor-associated macrophages as a paradigm for polarized M2 mononuclear phagocytes. Trends Immunol (2002) 23:549–55. 10.1016/S1471-4906(02)02302-5 12401408

[18] Sica A Bronte V. Altered macrophage differentiation and immune dysfunction in tumor development. J Clin Invest (2007) 117:1155–66. 10.1172/JCI31422 17476345 PMC1857267

[19] Mantovani A Sica A Locati M. Macrophage polarization comes of age. Immunity (2005) 23:344–6. 10.1016/j.immuni.2005.10.001 16226499

[20] Martinez FO Sica A Mantovani A Locati M Front macrophage activation and polarization. Front Biosci (2008) 13:453–61. 17981560 10.2741/2692

[21] Yong VW Chabot S Stuve O Williams G. Interferon beta in the treatment of multiple sclerosis: mechanisms of action. Neurology (1998) 51:682–9. 10.1212/WNL.51.3.682 9748010

[22] Van Weyenbergh J Wietzerbin J Rouillard D Barral-Netto M Liblau R. Treatment of multiplesclerosis patients with interferon-beta primes monocyte-derived macrophages for apoptotic cell death. J Leukoc Biol (2001) 70:745–8. 11698494

[23] Li Q Milo R Panitch H Swoveland P Bever CT Jr. Glatiramer acetate blocks the activation of THP-1 cells by interferon-gamma. Eur J Pharmacol (1998) 342:303–10. 10.1016/S0014-2999(97)01509-4 9548401

[24] Weber MS Prod’homme T Youssef S Dunn SE Rundle CD Lee L Type II monocytes modulate T cell-mediated central nervous system autoimmune disease. Nat Med (2007) 3:935–43. 10.1038/nm1620 17676050

[25] Ruggieri M Pica C Lia A Zimatore GB Modesto M Di Liddo E Combination treatment of glatiramer acetate and minocycline affects phenotype expression of blood monocyte-derived dendritic cells in multiple sclerosis patients. J Neuroimmunol (2008) 197:140–6. 10.1016/j.jneuroim.2008.04.032 18555539

[26] Collo G Neidhart S Kawashima E Kosco-Vilbois M North RA Buell G. Tissue distribution of the P2X7 receptor. Neuropharmacology (1997) 36:1277–83. 10.1016/S0028-3908(97)00140-8 9364482

[27] Di Virgilio F. The P2Z purinoceptor: an intriguing role in immunity, inflammation and cell death. Immunol Today (1995) 16:524–8. 10.1016/0167-5699(95)80045-X 7495489

[28] Caragnano M Tortorella P Bergami A Ruggieri M Livrea P Specchio LM Monocytes P2X7 purinergic receptor is modulated by glatiramer acetate in multiple sclerosis. J Neuroimmunol (2012) 245:93–7. 10.1016/j.jneuroim.2012.02.002 22370183

[29] Bianco F Perrotta C Novellino L Francolini M Riganti L Menna E Acid sphingomyelinase activity triggers microparticle release from glial cells. EMBO J (2009) 28(8):1043–54. 10.1038/emboj.2009.45 19300439 PMC2664656

[30] Di Virgilio F. Liaisons dangereuses: P2X(7) and the inflammasome. Trends Pharmacol Sci (2007) 28(9):465–72. 10.1016/j.tips.2007.07.002 17692395

[31] Gris D Ye Z Iocca HA Wen H Craven RR Gris P NLRP3 plays a critical role in the development of experimental autoimmune encephalomyelitis by mediating Th1 and Th17 responses. J Immunol (2010) 185(2):974–81. 10.4049/jimmunol.0904145 20574004 PMC3593010

[32] Inoue M Williams KL Gunn MD Shinohara ML. NLRP3 inflammasome induces chemotactic immune cell migration to the CNS in experimental autoimmune encephalomyelitis. Proc Natl Acad Sci U S A (2012) 109(26):10480–5. 10.1073/pnas.1201836109 22699511 PMC3387125

[33] Guarda G Braun M Staehli F Tardivel A Mattmann C Förster I Type I interferon inhibits interleukin-1 production and inflammasome activation. Immunity (2011) 34(2):213–23. 10.1016/j.immuni.2011.02.006 21349431

[34] Sheremata WA Jy W Delgado S Minagar A McLarty J Ahn Y. Interferon-beta1a reduces plasma CD31+ endothelial microparticles (CD31+EMP) in multiple sclerosis. J Neuroinflammation (2006) 3:23. 10.1186/1742-2094-3-23 16952316 PMC1584221

[35] Jimenez J Jy W Mauro LM Horstman LL Ahn ER Ahn YS Elevated endothelial microparticle-monocyte complexes induced by multiple sclerosis plasma and the inhibitory effects of interferon-beta 1b on release of endothelial microparticles, formation and transendothelial migration of monocyte-endothelial microparticle complexes. Mult Scler (2005) 11(3):310–5. 10.1191/1352458505ms1184oa 15957513

[36] Xu L Hilliard B Carmody RJ Tsabary G Shin H Christianson DW Arginase and autoimmune inflammation in the central nervous system. Immunology (2003) 110(1):141–8. 10.1046/j.1365-2567.2003.01713.x 12941151 PMC1783013

[37] Malmeström C Axelsson M Lycke J Zetterberg H Blennow K Olsson BJ. CSF levels of YKL-40 are increased in MS and replaces with immunosuppressive treatment. J Neuroimmunol (2014) 269(1–2):87–9. 10.1016/j.jneuroim.2014.02.004 24582001

[38] Ponomarev ED Maresz K Tan Y Dittel BN. CNS-derived interleukin-4 is essential for the regulation of autoimmune inflammation and induces a state of alternative activation in microglial cells. J Neurosci (2007) 27(40):10714–21. 10.1523/JNEUROSCI.1922-07.2007 17913905 PMC6672829

[39] Fairweather D Cihakova D. Alternatively activated macrophages in infection and autoimmunity. J Autoimmun (2009) 33(3–4):222–30. 10.1016/j.jaut.2009.09.012 19819674 PMC2783278

[40] Garzetti L Menon R Finardi A Bergami A Sica A Martino G Activated macrophages release microvesicles containing polarized M1 or M2 mRNAs. J Leukoc Biol (2014) 95(5):817–25. 10.1189/jlb.0913485 24379213

